# Long-term Clinical Outcomes of Posterior Spinal Stabilization Using Adeolu's Technique: A Prospective Study from Nigeria

**DOI:** 10.4314/ejhs.v34i5.9

**Published:** 2024-09

**Authors:** TB Rabiu

**Affiliations:** 1 Department of Surgery, UNIOSUN Teaching Hospital & Osun State University, Osogbo, Nigeria

**Keywords:** a. spinal stabilization, b. vertical struts, c. spinal process wires, d. Adeolu's technique, e. Nigeria, f. developing country

## Abstract

**Background:**

Due to resource constraints, advanced spinal stabilization methods such as pedicle screws are unavailable at our center. Adeolu's technique, which employs low-cost and readily available vertical struts and spinal process wires, has been used as an adjunct in treating various spinal conditions to achieve rigid spinal constructs. This study evaluates the long-term clinical outcomes of this technique.

**Methods:**

Nineteen patients treated with Adeolu's technique were prospectively followed to assess clinical outcomes. Primary outcomes included symptom reduction, absence of adverse events or need for re-intervention, and radiological evidence of stabilization and unrestricted mobilization. Secondary outcomes included back pain intensity, implant-related complications (rotation, migration, back-out, fracture), wound infection, worsening neurological status, and need for implant removal Data were analyzed using SPSS with simple descriptive statistics.

**Results:**

The average follow-up period was 4.1 years (Range: 0.1 – 9.0 years). Patients' ages ranged from 20 to 81 years (Mean: 48.5). Indications for surgery included lumbar spondylosis (7, 36.8%), spinal trauma with unstable fractures (4, 21.1%), spinal tumors (4, 21.1%), and Pott's disease (4, 21.1%). All patients achieved satisfactory primary outcomes. Implant rotation was observed in 4 patients (21.1%), and implant migration in 1 patient (5.3%), requiring removal. Superficial surgical site infection occurred in 2 patients (10.5%). No implant fractures, deep infections, or worsening neurological status were noted.

**Conclusion:**

Adeolu's technique is effective for a range of spinal pathologies, with favorable long-term clinical outcomes.

## Introduction

The cost of instrumented spine surgery is rising globally due to demographic changes and the adoption of new technologies (1,2). In developing countries, the use of advanced techniques and technologies is limited by both economic constraints and the lack of necessary intraoperative imaging facilities (3). Moreover, the transfer of equipment to low-income countries is often impeded by maintenance issues and environmental factors (4).

Hospitals in resource-limited settings frequently rely on donated implants and equipment, which are insufficient for the needs of all patients and create technical problems when complications arise (5). Therefore, the use of cost-effective and simple implants is essential in such settings (5). The introduction of Adeolu's technique, utilizing affordable and locally available materials, addresses these needs effectively (6).

Attempts to introduce locally-made or inexpensive alternatives to conventional spinal instrumentation have demonstrated comparable results to high-cost methods at reduced costs (7, 8, 9). Adeolu's technique, employing spinous process wiring with vertical struts, has proven to be a viable alternative to standard pedicle screws and rods for spinal stabilization (10, 11). This study evaluates the long-term clinical outcomes of spinal stabilization using Adeolu's technique in a cohort of patients managed in a university hospital.

## Methods

**Study design and patients' demographics:** This study included patients who underwent spinal stabilization using Adeolu's technique at UNIOSUN Teaching Hospital, Osogbo, Nigeria, between July 2012 and June 2022. A prospective database recorded patient demographics, clinical diagnoses, operative details, and follow-up outcomes.

**Operative procedures**: Adeolu's technique involves the use of vertical struts and spinal wires. Procedures were performed under general anesthesia with patients in the prone position. Laminectomies were performed as needed, and spinal wires were looped through holes at the bases of the spinous processes. Rush nails were secured with twisted wires to achieve rigid stabilization.

**Radiological studies**: Pre-operative X-rays and MRI were used to plan and assess the surgery. Post-operative X-rays were taken to verify implant placement and evaluate spinal stabilization over time.

**Primary outcomes**: Primary outcomes included symptom reduction, absence of adverse events, and evidence of rigid stabilization and unrestricted mobilization.

**Secondary outcomes**: Secondary outcomes included back pain intensity (measured by the visual analogue scale), implant complications, wound infections, and neurological status.

**Data analysis**: Descriptive statistical analyses were performed using SPSS version 26 (IBM Corp. 2019), and results were presented in text and tables.

## Results

The clinical profiles of the study subjects are set out in Table 1 and the summary of diagnoses and complications are provided in Tables 2 and 3 respectively..

**Table 1:** Clinical profile of study subjects

S/N	Sex	Age (Years)	Diagnosis	Type of Surgery	Duration of follow-up (Years)	Clinica outcome
1	F	44.0	Pott's disease	T7/T8 Laminectomies + drainage of spinal abscess + T5-T10 stabilization	4.0	Superficial Surgical Site Infection
2	F	43.0	Pott's disease	T6 Laminectomy + T4-T8 stabilization	3.5	
3	F	47.0	Pott's disease	T11/T12 Laminectomies + T9-L2 stabilization	0.3	
4	M	42.0	Pott's disease	T10/T11 Laminectomies + T8-L1 stabilization	0.1	
5	M	50.0	Lumbar Spondylosis + canal stenosis	L4 Laminectomy + L2-S1 stabilization	8.0	Implant rotation
6	M	43.0	Lumbar Spondylosis + L5 retrolisthesis	L4 Laminectomy + L2-S1 stabilization	9.0	Superficial Surgical Site Infection
7	F	60.0	Lumbar Spondylosis + canal stenosis	L2-5 Laminectomy + T12-S2 stabilization	7.0	Implant rotation
8	M	45.0	Lumbar Spondylosis + L2 paraplegia	L3 Laminectomy + L1-L5 stabilization	4.0	
9	F	52.0	Lumbar Spondylosis + canal stenosis	L4 Laminectomy + L2-S1 stabilization	2.0	
10	F	65.0	Lumbar Spondylosis + canal stenosis	L4/5 Laminectomies + L2-S2 stabilization	8.0	
11	M	75.0	Lumbar Spondylosis + canal stenosis	T11,L3/L5 Laminectomies + L1-S2 stabilization	0.3	
12	M	40.0	Traumatic L2 paraplegia	L2 Laminectomy + T12-L4 stabilization	8.0	
13	F	31.0	Traumatic T10 paraplegia	T12/L1 Laminectomies + T10-L3 stabilization	5.0	Implant rotation
14	M	62.0	Compression fracture of L1	T12/L1 Laminectomies + T10-L3 stabilization	6.0	
15	M	23.0	Traumatic T10 paraplegia	T12 Laminectomy + T10-L2 stabilization	5.0	
16	M	51.0	Prostate Carcinoma + spine metastasis	T5 &L2 Laminectomies + T3-T7 & T12-L4 stabilization	3.0	Implant migration at 4 years (removed)
17	F	20.0	L1 Vertebral tumour	L1 vertebrectomy + T11-L3 stabilization	3.5	
18	M	81.0	Prostate Carcinoma + spine metastasis	T12 Laminectomy + T10-L2 stabilization	0.5	
19	F	48.0	Breast carcinoma + Spine metastasis	T10/T11 Laminectomies + T8-L1 stabilization	0.5	Implant rotation

**Table 2:** Spectrum of diagnosis

Diagnosis	Number	Percent
Lumbar Spondylosis	7	36.8
Spine Trauma	4	21.1
Thoracic	(1)	(25.0)
Thoracolumbar	(2)	(50.0)
Lumbar	(1)	(50.0)
Pott's Disease	4	21.1
Spine tumour	4	21.1
Metastases	(3)	(75.0)
Carcinoma of the prostate	{2}	{66.7}
Breast Cancer	{1}	{33.3}
Primary bone tumour	(1)	(25.0)

**Table 3:** Complications

Complication	Number	Percent
Implant Rotation	4	21.1
Implant Fracture	0	0.0
Implant Migration	1	5.3
Surgical Site Infection (SSI)	0	0.0
Superficial	2	10.5
Deep	0	0.0
Worsening Neurologic Status	0	0.0

**Demographics**: Nineteen patients underwent the procedure, with an average age of 48.5 years (range: 20-81). The majority were in their fifth decade of life (32.1%).

**Diagnoses**: Indications for surgery included lumbar spondylosis (36.8%), spinal trauma (21.1%), spinal tumors (21.1%), and Pott's disease (21.1%).

**Follow-up**: Patients were followed for 2 months to 9 years, with an average follow-up period of 4.1 years.

**Figure 1:**
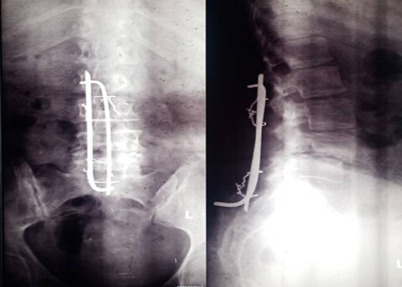
Example of implant rotation (on lateral view)

**Long-term outcomes**: All patients achieved satisfactory primary outcomes. Fifteen patients (78.9%) reported pain resolution, and 4 (21.1%) significantly reduced pain. Implant rotation was observed in 4 patients (21.1%) (an example is Figure 1), and one case of implant migration (5.3%) required removal. Superficial surgical site infection occurred in 2 patients (10.5%). No implant fractures, deep infections, or neurological deterioration were noted.

## Discussion

Spinal stabilization using vertical struts and spinous process wires made of stainless steel (Adeolu's technique) has been used by the author in the management of a variety of spinal diseases over the last decade. The implants are cheap and readily available in our society. This study showed that the long-term outcomes in the cohort of patients evaluated are good with acceptable low complication rates during a follow-up period of almost one decade.

The need for alternatives to conventional but very costly neurosurgical instruments, equipment and technologies has been highlighted by many authors(6-8,12,13). Similarly, other low-cost equipment has been reported to provide results comparable to higher-cost conventional ones in patient care (5). While the local alternatives in spinal instrumentation may appear on the surface to be inferior to the costly ones, many of them have been shown to have results that are comparable to the high-cost implants and technologies (14).

The utility of Adeolu's technique in managing spinal pathologies has been reported in many studies (3,7,10,11). The technique has proved simple to perform with low complication rates and good clinical outcomes in the short- and long term (3,7,10,11). The current study further demonstrates the satisfactory clinical profiles of the technique over a long follow-up period.

Degenerative spine disease, trauma, tumour and Pott's disease which were the indications for spinal stabilization in this study are similar to the indications/diagnoses in similar studies on spinal stabilization using this and similar techniques (3,7,11,15). Degenerative spine disease has also been found to be the most prevalent pathology in neuroimaging of the cervical spine (16).

The clinical outcomes noted in this study are similar to those observed in the initial short-term evaluation of the technique by this author and the long-term assessment provided by Adeolu and his colleague (3,11). However, as opposed to the findings by Adeolu et al., no patient had post-operative instability or spinous process fracture and none had post-operative neurologic deterioration.

Implant rotation remains a challenging outcome as the rate found in this study is slightly higher than that earlier reported in the short-term evaluation (3). This calls attention to the need to develop appropriate instrumentation for this technique. We currently use Kocher's forceps to twist the spinous process wires while tightening the Rush nails into place. The use of wire twisters or other appropriate instruments may limit the occurrence of this complication.

The low infection rate found in this study, which is similar to those of earlier reports is particularly gratifying (3,11). Furthermore, the infections responded well to antibiotics and wound dressing without the need for implant removal as also noted in the study from another Nigerian centre on the technique (11).

The patient in whom the implant was removed became debilitated most likely from the effect of the underlying malignancy which he had. His subsequent development of decubitus ulcer on the back led to the exposure of the implants which therefore had to be removed.

A major limitation of this study, as noted in the initial evaluation of the early outcomes of the technique, is that tests of in-vivo biomechanical strengths of the implants were not conducted in our patients (3). However, the non-occurrence of implant fracture and post-operative neurologic deterioration in this study supports the observation that the implants retain their biomechanical strengths for a long period and achieve the set-out objective of rigid spinal constructs in the wide variety of clinical conditions in which they were employed.

In conclusion, this study provided an evaluation of the long-term outcomes of Adeolu's techniques in a cohort of neurosurgical patients in a developing country with resource constraints. The technique offers utility in a variety of spinal pathologies and the long-term clinical outcomes are good.
